# A Risk Score with Additional Four Independent Factors to Predict the Incidence and Recovery from Metabolic Syndrome: Development and Validation in Large Japanese Cohorts

**DOI:** 10.1371/journal.pone.0133884

**Published:** 2015-07-31

**Authors:** Masaru Obokata, Kazuaki Negishi, Yoshiaki Ohyama, Haruka Okada, Kunihiko Imai, Masahiko Kurabayashi

**Affiliations:** 1 Department of Medicine and Biological Science, Gunma University Graduate School of Medicine, Maebashi, Gunma, Japan; 2 Clinical Investigation and Research Unit, Gunma University Graduate School of Medicine, Maebashi, Gunma, Japan; 3 Japan Community Healthcare Organization, Gunma Chuo Hospital, Maebashi, Gunma, Japan; 4 Menzies Institute for Medical Research, University of Tasmania, Hobart, Tasmania, Australia; Temple University School of Medicine, UNITED STATES

## Abstract

**Background:**

Although many risk factors for Metabolic syndrome (MetS) have been reported, there is no clinical score that predicts its incidence. The purposes of this study were to create and validate a risk score for predicting both incidence and recovery from MetS in a large cohort.

**Methods:**

Subjects without MetS at enrollment (n = 13,634) were randomly divided into 2 groups and followed to record incidence of MetS. We also examined recovery from it in rest 2,743 individuals with prevalent MetS.

**Results:**

During median follow-up of 3.0 years, 878 subjects in the derivation and 757 in validation cohorts developed MetS. Multiple logistic regression analysis identified 12 independent variables from the derivation cohort and initial score for subsequent MetS was created, which showed good discrimination both in the derivation (c-statistics 0.82) and validation cohorts (0.83). The predictability of the initial score for recovery from MetS was tested in the 2,743 MetS population (906 subjects recovered from MetS), where nine variables (including age, sex, γ-glutamyl transpeptidase, uric acid and five MetS diagnostic criteria constituents.) remained significant. Then, the final score was created using the nine variables. This score significantly predicted both the recovery from MetS (c-statistics 0.70, p<0.001, 78% sensitivity and 54% specificity) and incident MetS (c-statistics 0.80) with an incremental discriminative ability over the model derived from five factors used in the diagnosis of MetS (continuous net reclassification improvement: 0.35, p < 0.001 and integrated discrimination improvement: 0.01, p<0.001).

**Conclusions:**

We identified four additional independent risk factors associated with subsequent MetS, developed and validated a risk score to predict both incident and recovery from MetS.

## Introduction

Metabolic syndrome (MetS) is a growing public health issue that is becoming hyper-endemic around the world [1], with related increases in healthcare use and cost [2]. Unfortunately, however, the public’s recognition of MetS remains limited [3]. Although each constituent of MetS (i.e. elevated waist circumference, elevated triglycerides, reduced high-density lipoprotein cholesterol [HDL-C], elevated blood pressure or elevated fasting glucose) is known to be an independent contributor to cardiovascular disease, the clustering of these factors is also independently associated with an increased risk of adverse cardiovascular outcomes [4].

Interestingly, while obesity is less common in Asian populations compared to other ethnicities, the prevalence of MetS has been increasing in Asian countries [5, 6] and among Asian immigrants [7]. As the items used to diagnose MetS are modifiable risk factors, identifying the independent risk factors for MetS incidence, along with the subsequent risk stratification, would help to increase the public’s risk perception and motivate them to adopt healthier behaviors. Although many positively and inversely related risk factors have been reported [8–12], there is paucity of data regarding a predictive score for MetS. On the other hand, recent studies have shown that resolution from MetS has beneficial effect on atherosclerosis markers and diabetes mellitus [13, 14]. Thus, the aims of the present study were: 1) to identify the independent factors that predict incident MetS (independent from the items that are used to diagnose MetS), 2) to elucidate the magnitude of their predictive ability in a multivariate model, 3) to create a composite score to predict incident MetS, 4) to evaluate the predictive ability for recovery from MetS and update it, and 5) to assess this score’s discrimination and accuracy.

## Methods

### Study population

**Fig 1:**
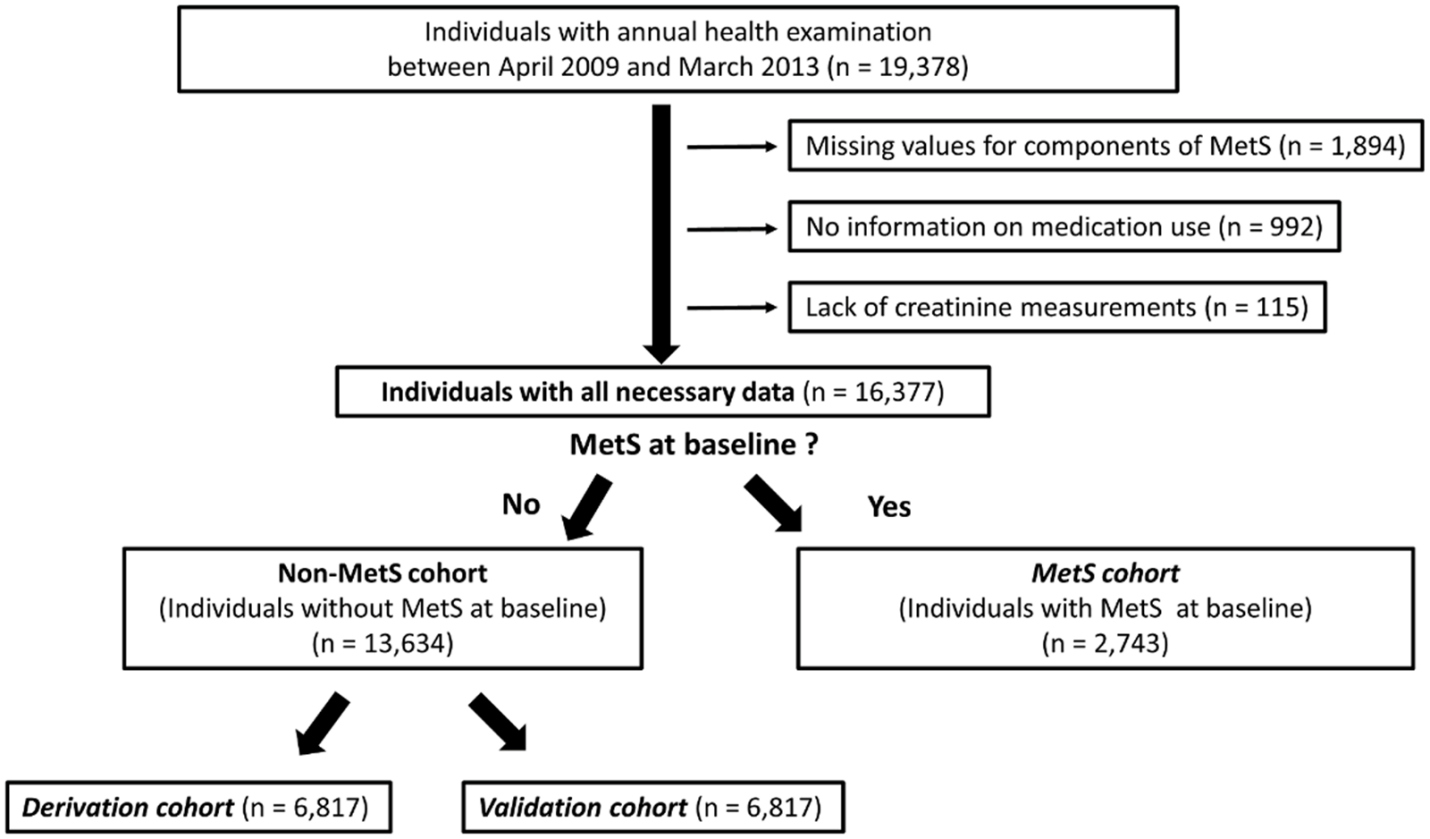
The study flow chart. MetS: metabolic syndrome.

We used the annual health examination database of Japanese employees and community-dwelling subjects who were evaluated at the Japan Community Healthcare Organization Gunma Chuo Hospital (Maebashi, Japan). In Japan, the Ordinance of the Ministry of Health, Labour, and Welfare requires that employers must ensure their workers receive annual medical examinations from a physician. The examination includes a health questionnaire, anthropometric measurements, blood tests, and a physical examination. As shown in Fig 1, we retrospectively screened 19,378 individuals ≥20 years old who received their annual examination at the hospital between April 2009 and March 2010.

We included all participants who had completed the measurements for each metabolic syndrome criteria at their index examination (defined as baseline), and who subsequently received at least one annual examination through 2013. As the Joint Interim Statement [15] classifies the use of specific drugs as equal criteria for meeting the definition of the MetS components, participants without detailed information regarding their medication use (n = 992) were excluded. We also excluded participants without data regarding their serum creatinine levels (n = 115), and ultimately included data from 13,634 participants without MetS and 2,743 individuals with MetS at baseline. The non-MetS study cohort was randomly divided into a derivation cohort (50% of the participants) to generate our initial model, and a validation cohort (50% of participants) to validate the model. The institutional ethics review boards of Gunma University Hospital (26–47) and Gunma Chuo Hospital (2014–004) approved this study with waiver of consent.

### Data collection

Participants completed a questionnaire that included questions regarding their medical history, lifestyle, and drug regimens. Anthropometric measurements included body weight, height, and waist circumference. Body mass index (BMI) was calculated by dividing the participant’s weight (kg) by the square of their height (m). Waist circumference was measured at the umbilicus during minimal respiration. After a brief period of quiet sitting, systolic and diastolic blood pressures (BP) were carefully measured by well-trained nurses. We collected blood samples after overnight fasting (>12 h), and the serum lipid, glucose, uric acid, and creatinine levels were measured using enzymatic methods.

### Definition of MetS

MetS was defined based on the criteria [15–18] from the Joint Interim Statement of the International Diabetes Federation Task Force of Epidemiology and Prevention; National Heart, Lung, and Blood Institute; American Heart Association; World Heart Federation; International Atherosclerosis Society; and International Association for the Study of Obesity. These criteria indicate that MetS is present if at least three of the following factors are present: (1) abdominal obesity: waist circumference ≥90 cm (Asian men) or ≥80 cm (Asian women); (2) hypertension: systolic BP ≥130 mmHg, diastolic BP ≥85 mmHg, or anti-hypertensive drug treatment; (3) elevated triglycerides: serum triglycerides ≥150 mg/dL or drug treatment for elevated triglycerides; (4) reduced HDL-C: HDL-C <40 mg/dL (men) or <50 mg/dL (women), or the use of medications to reduce HDL-C; and (5) hyperglycemia: fasting plasma glucose ≥100 mg/dL or drug treatment for elevated glucose.

### Statistical analyses

Continuous variables are presented as mean ± SD, unless otherwise specified. Categorical variables are expressed as frequency and percentage. The normality of the data was evaluated using the Kolmogorov-Smirnov test. Comparison of continuous variables between participants with and without incident metabolic syndrome were performed using Student’s *t* test for normally distributed data and the Mann-Whitney *U* test for non-normally distributed data. Categorical data were compared with Fisher’s exact test. Univariate and multivariate logistic regression analyses were used to determine the risk factors for incident MetS, and the association of potential risk factors with incident MetS was evaluated in the derivation cohort. Stepwise multiple logistic regression analyses, using Akaike information criteria for the model selection, were used to identify independent predictors of MetS incidence with continuous variables were dichotomized using an optimal cut-off points derived from Youden index, to construct a simple, general-purpose, easily implemented scoring system. The distribution of ages of all individuals in each MetS group is shown in S1 Fig.

These independent predictors were then assigned weighted points, which were proportional to their beta regression coefficient values [19, 20], and an initial risk score was calculated for each participant in the derivation cohort. The score was then applied to the validation cohort. C-statistics were used to determine the predictive ability of this score, and plots of the predicted risk vs. the observed risk outcomes were used to evaluate the score’s calibration. These analyses were performed in both the derivation and validation cohorts. Then, we determined which variable among them could predict recovery from MetS. Final score was created based on the variables selected in this model. The score was then refitted for the entire non-MetS population (n = 13,634), and the statistical significance of the difference in the area under the curves was compared using paired analyses [21].

Furthermore, the increased discriminative value was also assessed using the net reclassification improvement (NRI) and integrated discrimination improvement (IDI) [22]. The NRI examines the changes in estimated prediction probabilities, which imply a change from one category to another, between different models. In this analyses, we classified the probability of the risk into tertile (<2.0% [low], 2.0 to 9.0% [intermediate], and >9.0% [high]). The IDI is equal to the increase in the discrimination slope, which is defined as the difference between the mean of the estimated prediction probabilities (taken as continuous variables) for individuals with events and the corresponding mean for individuals without events. The continuous NRI was also used, which is a non-parametric analogue of the IDI that is equal to twice the difference in the probabilities of upward reclassification for events minus that for non-events [23]. For all analyses, two-tailed p-values were reported and p-values < 0.05 were considered statistically significant. All data were analyzed using SPSS version 21.0 (SPSS Inc., Chicago, IL, USA), MedCalc version 14.8.1 (MedCalc Software, Mariakerke, Belgium), and R version 3.1.0. (The R Foundation for Statistical Computing, Vienna, Austria) with the ‘PredictABEL’ package.

## Results

### Baseline characteristics of the derivation cohort

A total of 6,817 participants were included in the derivation cohort, and their baseline clinical characteristics are shown in Table 1. Participants who developed MetS were older and more obese, compared to participants who did not develop MetS. In addition, male sex and daily alcohol consumption were more common in the MetS group. Furthermore, baseline waist circumference, systolic and diastolic BP, liver enzymes, triglycerides, low-density lipoprotein cholesterol (LDL-C), fasting glucose, uric acid, hematocrit, and hemoglobin levels were significantly higher among participants who developed MetS. In contrast, HDL-C levels and the estimated glomerular filtration rates were significantly lower among participants who developed MetS. Individuals who developed subsequent MetS were more likely to be receiving medications for hypertension, dyslipidemia, diabetes, and gout, compared to those who did not develop MetS. All five diagnostic constituents were more common among participants who developed MetS compared to those who did not develop MetS.

**Table 1:** Baseline Characteristics of the Development Cohort and Crude Association of Potential Risk Factors with Incident Metabolic Syndrome.

Characteristics	All subjects(n = 6817)	Incident MetS		P Value	Crude odds ratio(95% CI)
		No (n = 5939)	Yes (n = 878)		
Age, yrs	50.8 ± 8.8	50.4 ± 8.8	53.4 ± 8.2	<0.001*	1.04 (1.03–1.05)
Female gender	38.2%	2334 (39.3%)	271 (30.9%)	<0.001†	0.69 (0.59–0.80)
Body mass index, kg/m^2^	22.5 ± 2.8	22.2 ± 2.7	24.6 ± 3.0	<0.001*	1.33 (1.30–1.37)
Prior stroke	1.0%	51 (0.9%)	20 (2.3%)	<0.001†	2.69 (1.60–4.54)
Ischemic heart disease	2.1%	121 (2.0%)	22 (2.5%)	0.376†	1.24 (0.78–1.96)
Current smoking	30.5%	1789 (30.1%)	293 (33.4%)	0.054†	1.16 (1.00–1.35)
Daily alcohol	29.6%	1720 (29.0%)	295 (33.6%)	0.006†	1.24 (1.07–1.44)
Waist circumference, cm	80.2 ± 8.1	79.2 ± 7.8	86.3 ± 7.7	<0.001*	1.12 (1.11–1.13)
Systolic BP, mm Hg	124.0 ± 15.6	122.9 ± 15.3	131.3 ± 15.6	<0.001*	1.03 (1.03–1.04)
Diastolic BP, mm Hg	78.1 ± 10.8	77.3 ± 10.5	83.5 ± 10.9	<0.001*	1.05 (1.05–1.06)
Aspartate aminotransferase, IU/L	20.9 ± 8.9	20.5 ± 8.5	23.6 ± 10.7	<0.001*	1.03 (1.02–1.04)
Alanine aminotransferase, IU/L	21.2 ± 14.8	20.3 ± 14.1	27.2 ± 17.6	<0.001*	1.02 (1.02–1.03)
γ-GTP, IU/L	36.8 ± 42.4	34.9 ± 39.5	49.7 ± 56.6	<0.001*	1.01 (1.00–1.01)
Alkaline Phosphatase, IU/L	215.5 ± 61.4	213.0 ± 60.8	232.6 ± 62.5	<0.001*	1.01 (1.00–1.01)
Triglycerides, mg/dL	102.9 ± 64.0	97.1 ± 58.1	142.3 ± 84.8	<0.001*	1.01 (1.01–1.01)
HDL-C, mg/dL	66.0 ± 16.3	67.3 ± 16.4	57.6 ± 13.5	<0.001*	0.96 (0.95–0.96)
LDL-C, mg/dL	127.5 ± 31.4	125.4 ± 30.7	141.8 ± 32.4	<0.001#	1.02 (1.01–1.02)
Fasting glucose, mg/dL	92.8 ± 14.8	92.0 ± 14.3	97.7 ± 16.8	<0.001*	1.02 (1.02–1.02)
eGFR, ml/min/1.73m^2^	82.1 ± 13.5	82.5 ± 13.4	79.3 ± 13.8	<0.001*	0.98 (0.98–0.99)
Uric acid, mg/dL	5.3 ± 1.3	5.2 ± 1.3	5.8 ± 1.4	<0.001*	1.36 (1.29–1.44)
Red blood cell count, 10^4^/μL	469.3 ± 43.6	467.5 ± 43.3	481.1 ± 43.4	<0.001*	1.01 (1.01–1.01)
Hematocrit, %	43.1 ± 4.0	42.9 ± 4.0	44.2 ± 3.8	<0.001*	1.09 (1.07–1.11)
Hemoglobin, g/dL	14.2 ± 1.5	14.2 ± 1.5	14.7 ± 1.4	<0.001*	1.28 (1.21–1.34)
**Medications**					
Antihypertensive drugs	8.9%	432 (7.3%)	172 (19.6%)	<0.001†	3.11 (2.56–3.77)
Lipid lowering drugs	0.9%	42 (0.7%)	20 (2.3%)	<0.001†	3.27 (1.91–5.60)
Glucose lowering drugs	1.4%	67 (1.1%)	27 (3.1%)	<0.001†	2.78 (1.77–4.37)
Uric acid lowering drugs	0.7%	37 (0.6%)	14 (1.6%)	0.005†	2.59 (1.39–4.80)
**Metabolic components**					
Abdominal obesity	20.2%	1007 (17.0%)	371 (42.3%)	<0.001†	3.58 (3.08–4.16)
Hypertension	37.7%	2044 (34.4%)	523 (59.6%)	<0.001†	2.81 (2.43–3.25)
Elevated triglycerides	14.1%	703 (11.8%)	258 (29.4%)	<0.001†	3.10 (2.63–3.66)
Reduced HDL-C	3.3%	165 (2.8%)	63 (7.2%)	<0.001†	2.71 (2.01–3.65)
Hyperglycemia	15.1%	829 (14.0%)	198 (22.6%)	<0.001†	1.80 (1.51–2.14)

Comparisons between participants with and without incident metabolic syndrome using Student’s *t* test (#), Mann-Whitney *U* test (*), or Fisher’s exact test (†). BP indicates blood pressure; CI, confidential interval; eGFR, estimated glomerular filtration rate; GTP, glutamyl transpeptidase; HDL-C, high-density lipoprotein cholesterol; LDL-C, low-density lipoprotein cholesterol; MetS, metabolic syndrome.

### Incidence of MetS and construction of the risk score

**Fig 2:**
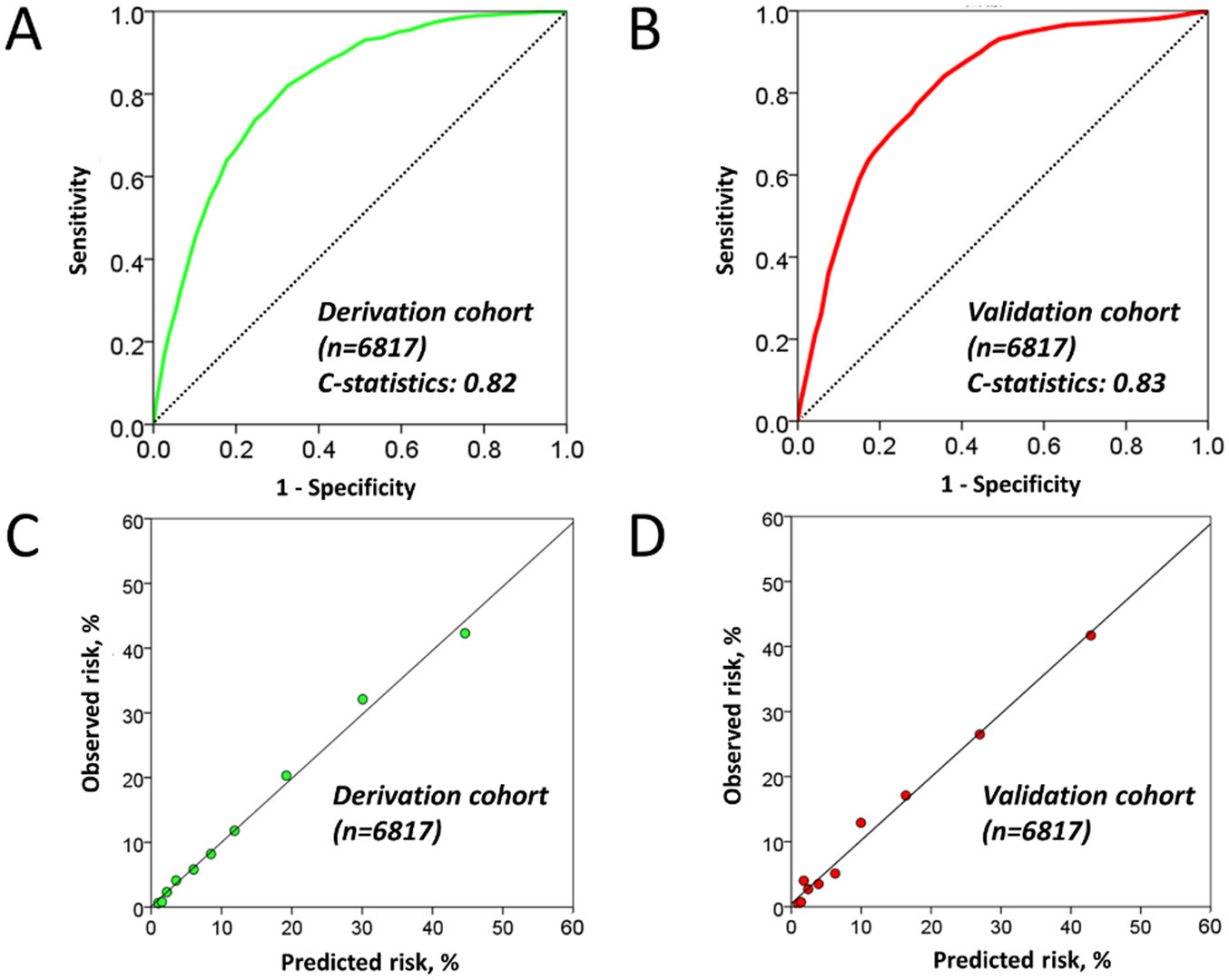
Receiver-operating characteristics curves and correlation of predicted versus observed risk of outcomes of an initial risk score for predicting Metabolic Syndrome in the derivation and validation cohorts. The initial score accurately predicted incident MetS both in the derivation **(A)** and validation cohorts **(B)**. Calibration plots for prediction of incident MetS in the derivation cohort **(C)** (Intercept of 0.12, a slope of 0.99, and an R^2^ of 99%, p <0.001) and validation cohorts **(D)** (Intercept of 0.48, a slope of 0.97, and an R^2^ of 99%, p <0.001). Abbreviations as in Fig 1.

During the median follow-up of 3.0 years (interquartile range: 2.8–3.0 years), 878 individuals in the derivation cohort developed MetS. Therefore, we used univariate logistic regression analyses to test the relationships between the potential risk factors and the incidence of MetS, and observed several significant associations with the risk of MetS (Table 1). We then performed the stepwise multivariate logistic regression analyses using Akaike information criteria, where continuous variables were dichotomized using their optimal cut-off for ease of use in the subsequent risk score (S1 Table). All the variables that were significantly associated with the risk of MetS in the univariate analysis, were subsequently entered into the multivariate model. In the stepwise multivariate analysis, 12 factors remained as predictors of MetS: age >47 years old; female gender; abdominal obesity; elevated triglycerides; reduced HDL-C; hypertension; hyperglycemia; alkaline phosphatase levels >200 IU/l; hematocrit >45%; LDL-C >130 mg/dl; γglutamyl-transpeptidase levels >30 IU/l; and uric acid levels > 6.0 mg/dl (Table 2). Of note, female gender was kept in the final model because inclusion of this yielded better (i.e. smaller) AICs although it was not independent. To construct an initial risk score, we assigned each of the 12 risk factors a weighted score (1–14 points) proportional to their beta regression coefficient values and each individual’s score was then calculated by adding the points for each factor (0 to 73 points). The initial score exhibited good discrimination (c-statistic: 0.82) in the derivation cohort (p < 0.001) (Fig 2A), and the observed vs. predicted risk of incident MetS within the risk deciles is shown in Fig 2C. The score exhibited good calibration with the observed events, with an intercept of 0.12, a slope of 0.99, and an R^2^ of 99%.

**Table 2:** Multivariate Logistic Regression Analysis for the Prediction of Metabolic Syndrome and Risk Scoring System in the Derivation cohort.

Variables	Odds Ratio (95% CI)	p Value	Beta Coefficient	Points
Abdominal obesity	5.82 (4.78–7.09)	<0.001	1.76	14
Reduced HDL-cholesterol	4.22 (2.98–5.98)	<0.001	1.44	11
Elevated triglycerides	3.97 (3.22–4.91)	<0.001	1.38	11
Hypertension	3.37 (2.82–4.04)	<0.001	1.22	10
Hyperglycemia	2.68 (2.17–3.31)	<0.001	0.99	8
LDL-cholesterol > 130 mg/dL	1.74 (1.47–2.06)	<0.001	0.55	4
Age > 47 years	1.67 (1.38–2.02)	<0.001	0.51	4
γ-GTP > 30 IU/L	1.48 (1.23–1.78)	<0.001	0.39	3
Uric acid > 6.0 mg/dL	1.39 (1.15–1.67)	0.001	0.33	3
Hematocrit > 45%	1.30 (1.07–1.57)	0.008	0.26	2
Alkaline Phosphatase > 200 IU/L	1.29 (1.08–1.54)	0.004	0.25	2
Female gender	1.14 (0.88–1.46)	0.321	0.13	1

Abbreviations as in Table 1.

During the same follow-up period, 757 subjects developed MetS in the validation cohort (n = 6,817). Similar c-statistic and good calibration were obtained when applying the score in the validation cohort (c-statistic: 0.83, p < 0.001) (Fig 2B and 2D).

### Development of a Risk Score for predicting recovery from MetS

**Fig 3:**
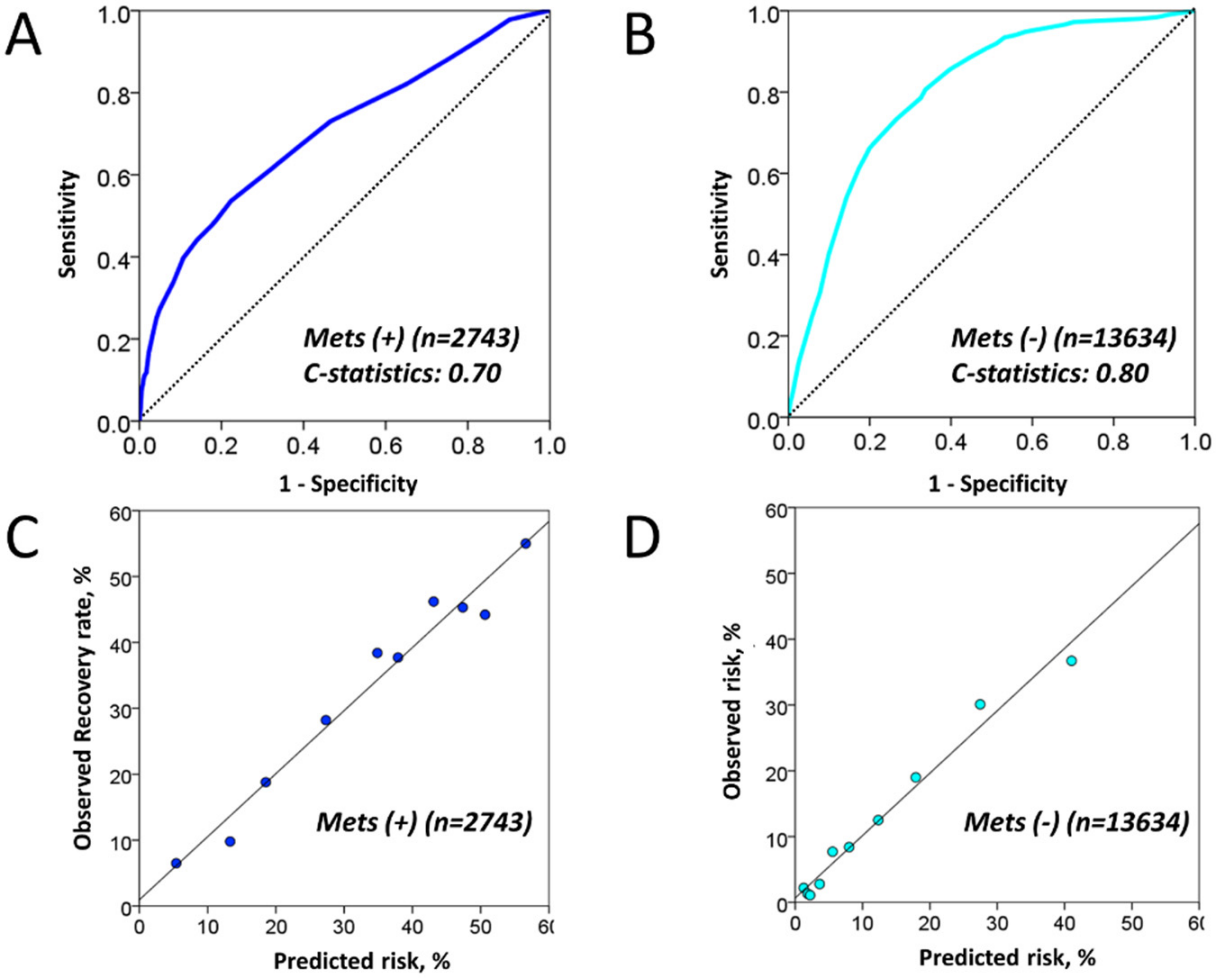
Receiver-operating characteristics curves and predicted risk versus observed risk of outcomes of a final risk score for predicting MetS and recovery from it. **(A)** In MetS cohort, the final risk model predicted the recovery from MetS. **(B)** The final score predicted subsequent MetS in the whole population without MetS at enrollment. **(C)** Calibration plots for prediction of recovery from MetS in the prevalent MetS population at enrollment were good with an intercept of 0.95, a slope of 0.96, and an R^2^ of 97% (p <0.001). **(D)** The final score demonstrated good calibration for predicting incident MetS in the whole population with an intercept of 0.69, a slope of 0.95, and an R^2^ of 98% (p <0.001). Abbreviations as in Fig 1.

**Fig 4:**
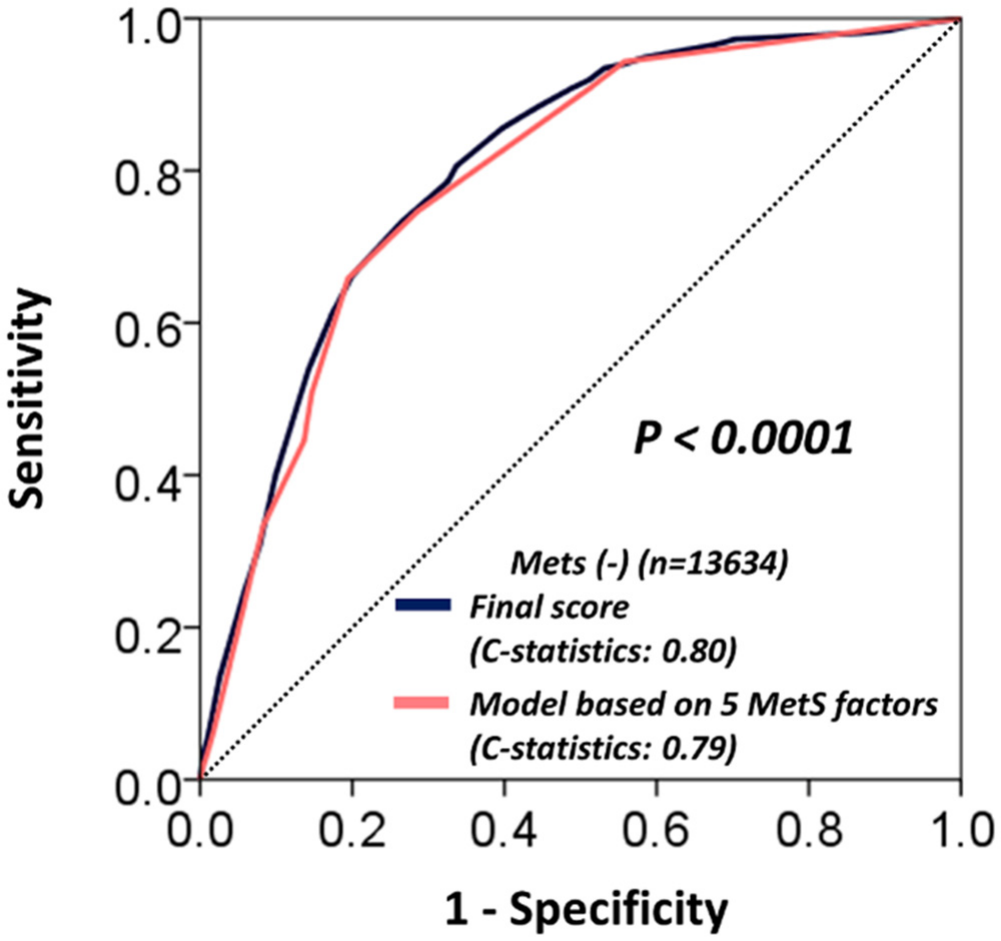
Comparison of area under the curves between final risk score and model derived from only the five MetS diagnostic components. The final score had a significantly larger area under the curve compared with the area obtained using the model that was derived from only the five MetS diagnostic components (0.80 vs. 0.79, p < 0.0001). Abbreviations as in Fig 1.

**Fig 5:**
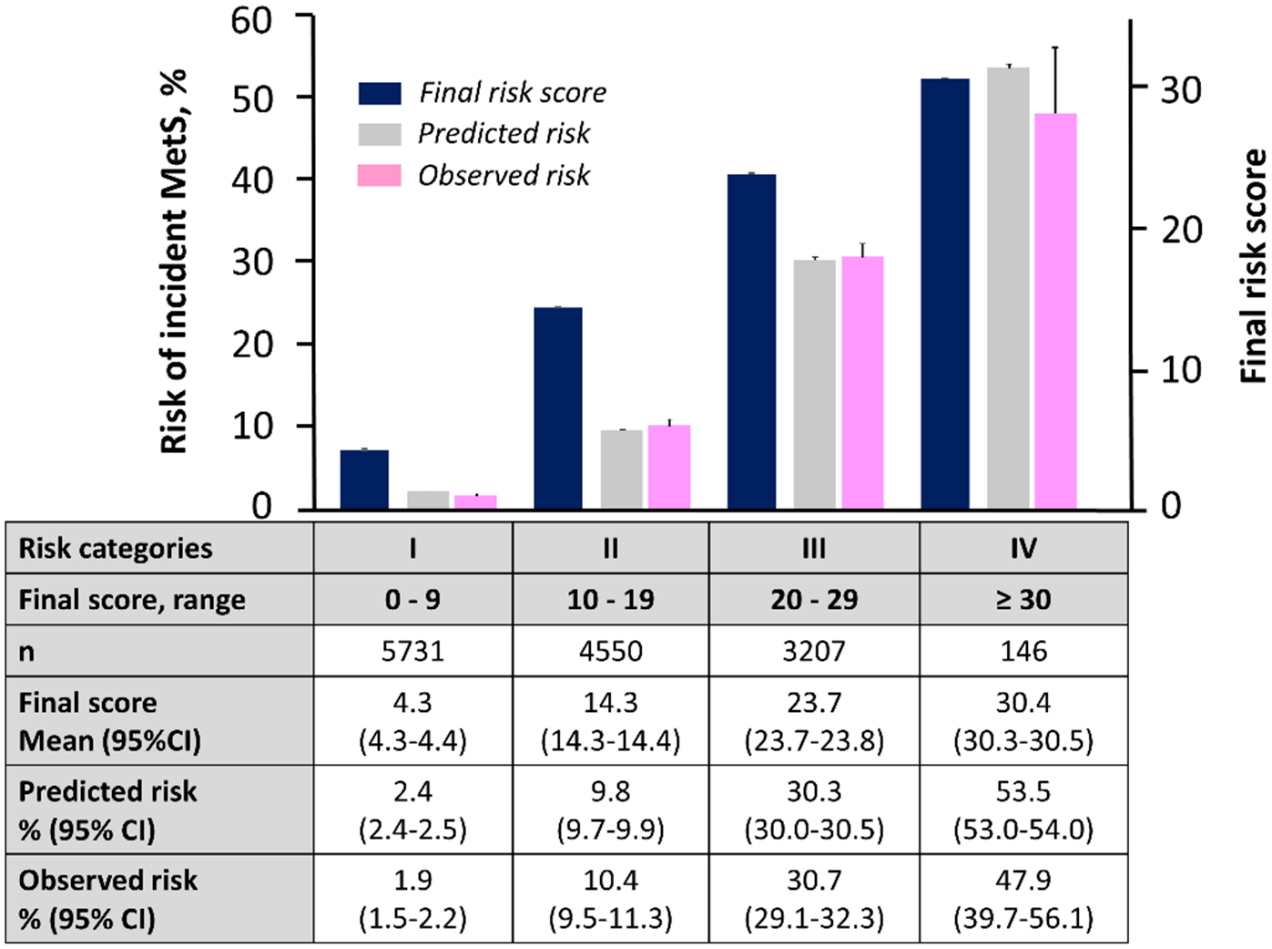
Final score predicts incidence of MetS in the entire Non-MetS population. Final risk scores for incident MetS were calculated for each individual participant in the entire population without MetS at enrollment (derivation and validation cohorts combined, n = 13,634) as described in Table 4. Incidence (%) in the bottom table represents the developed MetS cases in the population (n = 1,635). I bars represent 95% confidential interval (CI). Other abbreviations as in Fig 1.

We determined which variable among the initial risk score could predict recovery from MetS in order to identify risk factors more closely associated to MetS. In the MetS population at enrollment, 906 subjects recovered from it (Table 3). Among the 12 variables included in the initial score, hematocrit, LDL-C and alkaline phosphatase levels were not correlated with the recovery. Final score was then created using the rest nine variables which were re-assigned weighted points proportional to the beta regression coefficient values in the new multivariate logistic regression analysis (Table 4). The final score significantly predicted the recovery from MetS (c-statistics 0.70, p<0.001) and the score < 36 points identified the recovery with a 78% sensitivity and 54% specificity (Fig 3A). The final model also demonstrated good calibration for the recovery with an intercept of 0.95, a slope of 0.96, and an R^2^ of 97% (Fig 3C). When we refitted the final score in the entire population without MetS, the final score accurately predicted incident MetS (c-statistics 0.80, 95% CI [0.79, 0.81]) and the calibration remained good (Fig 3B and 3D). In addition, the final score accurately predicted for incident MetS when it was refitted to both the derivation and validation cohorts (c-statistics 0.79, 95% CI [0.78–0.81], p<0.001 and c-statistics 0.81, 95% CI [0.80–0.83], p<0.001, respectively). Importantly, the final score had a significantly larger area under the curve compared with the area obtained using the model that was derived from only the five MetS diagnostic components (0.80 vs. 0.79, p < 0.0001) (Fig 4). Furthermore, the risk score provided significant incremental discriminative ability compared to the model that was derived from only the five MetS diagnostic components, as assessed using the tertile-based NRI (0.34, 95% CI: 0.32, 0.36, p < 0.001) (S2 Table), the continuous NRI (0.35, 95% CI: 0.30, 0.41, p < 0.001), and the IDI (0.01, 95% CI: 0.01, 0.01, p<0.001). Lastly, the final score was applied to the entire non-MetS population (derivation and validation cohorts combined, n = 13,634) to assess its predictive ability for incident MetS. Fig 5 shows the distribution of the scores in the entire population, as well as the predicted incidence of MetS within the various score categories. Category I (scores: 0–9 points) had an incidence risk of 2.4% (95% CI: 2.4, 2.5). Category II (10–19 points) had an incident risk of 9.8% (95% CI: 9.7, 9.9), Category III (20–29 points) had an incidence risk of 30.3% (95% CI: 30.0, 30.5), and Category IV (≥ 30 points) had an incidence risk of 53.5% (95% CI: 53.0, 54.0).

**Table 3:** Baseline Characteristics of the MetS Population and Crude Association of Potential Risk Factors with Recovery from Metabolic Syndrome.

Characteristics	MetS subjects (n = 2743)	Recovery from MetS		P Value	Crude odds ratio** (95% CI)
		No (n = 1837)	Yes (n = 906)		
Age, yrs	52.4 ± 9.4	53.0 ± 9.3	51.3 ± 9.4	<0.001*	0.98 (0.97–0.99)
Female gender	24.2%	489 (26.6%)	175 (19.3%)	<0.001†	0.66 (0.54–0.80)
Body mass index, kg/m^2^	26.4 ± 3.6	26.7 ± 3.8	25.7 ± 3.1	<0.001*	0.92 (0.90–0.94)
Prior stroke	1.9%	39 (2.1%)	12 (1.3%)	0.176†	0.62 (0.32–1.19)
Ischemic heart disease	4.0%	95 (5.2%)	14 (1.5%)	<0.001†	0.29 (0.16–0.51)
Current smoking	35.8%	650 (35.4%)	332 (36.6%)	0.526†	1.06 (0.90–1.25)
Daily alcohol	30.7%	537 (29.2%)	304 (33.6%)	0.022†	1.22 (1.03–1.45)
Waist circumference, cm	91.5 ± 8.7	92.4 ± 9.0	89.8 ± 7.6	<0.001*	0.96 (0.96–0.97)
Systolic BP, mm Hg	138.9 ± 16.3	139.6 ± 16.8	137.5 ± 15.0	0.002*	0.99 (0.99–1.00)
Diastolic BP, mm Hg	87.6 ± 10.7	88.0 ± 11.1	86.8 ± 9.7	0.010*	0.99 (0.98–1.00)
Aspartate aminotransferase, IU/L	26.2 ± 14.1	26.7 ± 14.8	25.4 ± 12.6	0.100*	0.99 (0.99–1.00)
Alanine aminotransferase, IU/L	34.4 ± 26.2	35.3 ± 27.7	32.7 ± 22.8	0.048*	1.00 (0.99–1.00)
γ-GTP, IU/L	60.6 ± 63.6	60.6 ± 63.9	60.6 ± 63.1	0.587*	1.00 (1.00–1.01)
Alkaline Phosphatase, IU/L	234.2 ± 66.7	233.9 ± 67.8	234.8 ± 64.4	0.369*	1.00 (1.00–1.00)
Triglycerides, mg/dL	199.0 ± 166.7	203.0 ± 184.4	191.0 ± 122.9	0.947*	1.00 (1.00–1.00)
HDL-C, mg/dL	53.4 ± 13.8	53.2 ± 13.8	53.8 ± 13.8	0.338*	1.00 (1.00–1.01)
LDL-C, mg/dL	139.1 ± 33.9	139.2 ± 35.4	139.0 ± 30.7	0.881#	1.00 (1.00–1.00)
Fasting glucose, mg/dL	110.2 ± 30.5	112.0 ± 31.2	106.4 ± 28.8	<0.001*	0.99 (0.99–1.00)
eGFR, ml/min/1.73m^2^	80.9 ± 15.7	80.8 ± 16.2	81.2 ± 14.6	0.276*	1.00 (1.00–1.01)
Uric acid, mg/dL	6.0 ± 1.4	6.0 ± 1.4	6.1 ± 1.4	0.593*	1.02 (0.96–1.08)
Red blood cell count, 10^4^/μL	492.6 ± 43.4	492.2 ± 43.3	493.4 ± 43.5	0.276*	1.00 (1.00–1.00)
Hematocrit, %	45.0 ± 3.7	44.9 ± 3.7	45.1 ± 3.7	0.054*	1.02 (0.99–1.04)
Hemoglobin, g/dL	15.0 ± 1.4	14.9 ± 1.4	15.0 ± 1.4	0.014*	1.05 (0.99–1.11)
**Medications**					
Antihypertensive drugs	29.7%	661 (36.0%)	154 (17.0%)	<0.001†	0.36 (0.30–0.44)
Lipid lowering drugs	20.0%	498 (27.1%)	51 (5.6%)	<0.001†	0.16 (0.12–0.22)
Glucose lowering drugs	9.8%	232 (12.6%)	38 (4.2%)	<0.001†	0.30 (0.21–0.43)
Uric acid lowering drugs	3.8%	85 (4.6%)	20 (2.2%)	0.001†	0.47 (0.28–0.76)
**Metabolic components**					
Abdominal obesity	74.0%	1401 (76.3%)	628 (69.3%)	<0.001†	0.70 (0.59–0.84)
Hypertension	87.2%	1605 (87.4%)	787 (86.9%)	0.716†	0.96 (0.75–1.21)
Elevated triglycerides	76.6%	1435 (78.1%)	667 (73.6%)	0.010†	0.78 (0.65–0.94)
Reduced HDL-C	39.0%	845 (46.0%)	226 (24.9%)	<0.001†	0.39 (0.33–0.47)
Hyperglycemia	65.3%	1236 (67.3%)	554 (61.1%)	0.002†	0.77 (0.65–0.90)

Comparisons between participants with and without incident metabolic syndrome using Student’s *t* test (#), Mann-Whitney *U* test (*), or Fisher’s exact test (†).

**Odds ratios to predict recovery from Mets (e.g. Odd ratio<1 means less likely to be recovered subsequently).

Abbreviations as in Tables 1 and 2.

**Table 4:** Multivariate Logistic Regression Analysis for the Recovery from Metabolic Syndrome and Risk Scoring System in individuals with Prevalent Metabolic Syndrome.

Variables	Odds Ratio* (95% CI)	p Value	Beta Coefficient	Points
Reduced HDL-cholesterol	0.18 (0.14–0.23)	<0.001	-1.73	13
Abdominal obesity	0.28 (0.22–0.36)	<0.001	-1.28	10
Hyperglycemia	0.30 (0.23–0.38)	<0.001	-1.21	9
Hypertension	0.32 (0.24–0.43)	<0.001	-1.14	9
Elevated triglycerides	0.38 (0.30–0.50)	<0.001	-0.96	7
Female gender	0.61 (0.48–0.79)	<0.001	-0.49	4
γ-GTP > 30 IU/L	0.63 (0.51–0.77)	<0.001	-0.47	4
Age > 47 years	0.71 (0.59–0.85)	<0.001	-0.35	3
Uric acid > 6.0 mg/dL	0.88 (0.73–1.05)	0.157	-0.13	1

*Odds ratios to predict recovery from MetS (e.g. Odds ratio<1 means less likely to be recovered subsequently).

Other Abbreviations as in Tables 1 and 2.

## Discussion

In this observational study, we identified four independent predictors of incident MetS and developed a score to predict incident MetS among large Japanese cohort. As far as we know, this is the first study which created a prediction score for incident MetS after elucidating the predictability for recovery from MetS and updating it. The final risk score had good predictability, with an incremental discriminative value over the model derived using only the five diagnostic constituents. The score placed each individual subject into one of four risk categories, with a MetS incidence range between 2.4% to 53.5%.

Identification of the risk factors of MetS fits closely with the current AHA 2020 Impact Goals, where the prevalence of MetS is a secondary metric [24]. As in Western countries, the prevalence of MetS in Asian countries has been increasing over the past several decades. Thus, MetS can also become a larger global health issue. Our results indicate that abdominal obesity was the strongest contributor to incident MetS, followed by the remaining four diagnostic components (which had similar ORs). This finding is consistent with the fact that the predominant underlying factors for MetS appear to be abdominal obesity and insulin resistance [25]. On the other hand, Reduced HDL-cholesterol was the strongest determinant for the recovery from MetS. Although HDL-cholesterol is known as a risk factor for developing MetS in multiple ethnicities [26, 27], this is the first study which highlighted its importance from the resolution perspective. HDL modifying interventions, such as exercise and medications (statins, nicotinic acid and fibrates) can be considered for MetS population.

We also identified four additional risk factors for incident MetS (age, female sex, higher levels of uric acid and γ-glutamyl transpeptidase), which were independent of the five diagnostic components. Our reclassification analyses indicated that the predictability provided by the model including these four factors and the five MetS diagnostic components was incremental to the model using only the five MetS diagnostic components. Interestingly, previous data indicate that these risk factors are independently associated with incident MetS, as age is a well-known risk factor for MetS in both the USA [8] and Asian countries [10]. Growing evidence also suggests that body fat redistribution occurs during advancing age, with an increase in visceral fat and a relative loss of subcutaneous fat [28]. This fat redistribution is thought to be related to insulin resistance through chronic inflammation, leading to an increased risk of MetS [29]. We demonstrated that female sex was an independent risk for MetS. Although the effect of sex difference on the incidence of the syndrome remains uncertain, prevalence rates have increased especially in young women in the United States [30]. Emerging data demonstrate that heterogeneity between men and women, in part related to hormonal regulation of body fat distribution and influence of estrogen decline of risk factor clustering [31]. Prospective studies also show that elevated uric acid levels are associated with incident MetS independent from components of MetS [9, 32, 33]. It is likely that oxidative stress and inflammation would play a key role for the relationship. Previous data reported a potential role of uric acid as prooxidant, which induces the activation of proinflammatory state and oxidative stress in adipocytes [34]

Several investigations support our results that some liver enzymes were independently associated with increased risk of MetS. Among liver enzymes, γ-glutamyl-transpeptidase is the main predictor of diabetes mellitus incidence and may be a marker of insulin resistance. Recent investigations have reported that γ-glutamyl transpeptidase is associated with the risk of developing MetS, independent of excessive alcohol consumption and liver diseases [35]. Although the mechanisms underlying the association between γ-glutamyl transpeptidase and MetS have not been fully elucidated, the enzyme is associated with hepatic steatosis, non-alcoholic fatty liver disease, and may contribute to incident MetS via inflammation, oxidative stress pathways and insulin resistance [36].

As MetS should be considered largely a disease of unhealthy lifestyle [24], identification of the high-risk population of incident MetS can trigger an earlier action for the healthcare provider and patient to address the underlying lifestyle-related risk factors and to increase their risk perception and motivation to promote healthier behaviors. Although lifestyle modifications, such as increasing physical activity and body weight reduction, have potential as primary measures to prevent MetS, the current health examinations mainly assess patients for the presence of MetS to intervene. In contrast, our risk score accurately identified high risk individuals with MetS incident rates of 53.5% while they were in pre-MetS stage. Furthermore, the score predicted the recovery from MetS among individuals with prevalent MetS. It is worth emphasizing because resolution from MetS has beneficial effect on atherosclerosis markers. Early pharmacological treatment can be cost-effective in pre-MetS individuals, as healthcare costs increase by approximately 24% for each additional MetS component that is present [2]. Therefore, this score can be utilized to facilitate earlier lifestyle modifications and/or medical treatments.

### Study limitation

Our study has several limitations. First, the percentage of female in this study was lower than the natural female percentage in general population. Most of examinees in our database were employees and might not be representative of the general population. Second, the definition of MetS according to the Joint Interim Statement recommends that the cutoff values for abdominal obesity be based on a specific population or country. Therefore, it appears unlikely that our findings would be readily generalizable to other ethnic populations. However, a score for Asians is very important from the global standpoint because more than a half of world population is Asian ethnicity [37]. Third, we could not evaluate several important variables in this study, including dietary habits, physical activity, and menopausal status because this was a retrospective study. Fifth, although we validated the score in both validation and MetS cohort, they are internal validation. Thus, our score should be validated in external cohorts.

In conclusion, we identified four additional predictors of MetS, and developed a risk score to predict both incident and recovery from MetS in a large Japanese population. We believe this information can be used in public education, prevention initiatives, and the prevention and management of MetS.

## Supporting Information

S1 Fig The distribution of ages of all individuals in the derivation cohort. Age was dichotomized using optimal cut-off points (47 years) derived from Youden index to construct a scoring system. Pink bar: participants without MetS, dark-blue bar: participants with incident MetS; abbreviations as in Fig 1.
(TIF)

S1 Table Receiver-operating characteristic curve of parameters predicting incident metabolic Syndrome. Abbreviations as in Tables 1 and 2.
(DOCX)

S2 Table Reclassification tables for NRI calculation for subsequent MetS risk. Reclassification was assessed using these two tables: those with subsequent MetS (*Top*) and those without (*Bottom*). Although 209 people were misclassified in the top table, 5646 people without outcome (subsequent Mets) were correctly reclassified into lower risk category. Consequently, 34% of Non-MetS population were correctly reclassified. NRI, net reclassification improvement; other abbreviations as in Table 1.
(DOCX)
