# Supplementary figures and images for: A Risk Score with Additional Four Independent Factors to Predict the Incidence and Recovery from Metabolic Syndrome: Development and Validation in Large Japanese Cohorts
Source: PLoS One. 2015 Jul 31;10(7):e0133884. doi: 10.1371/journal.pone.0133884 (PMC4521863; doi:10.1371/journal.pone.0133884)

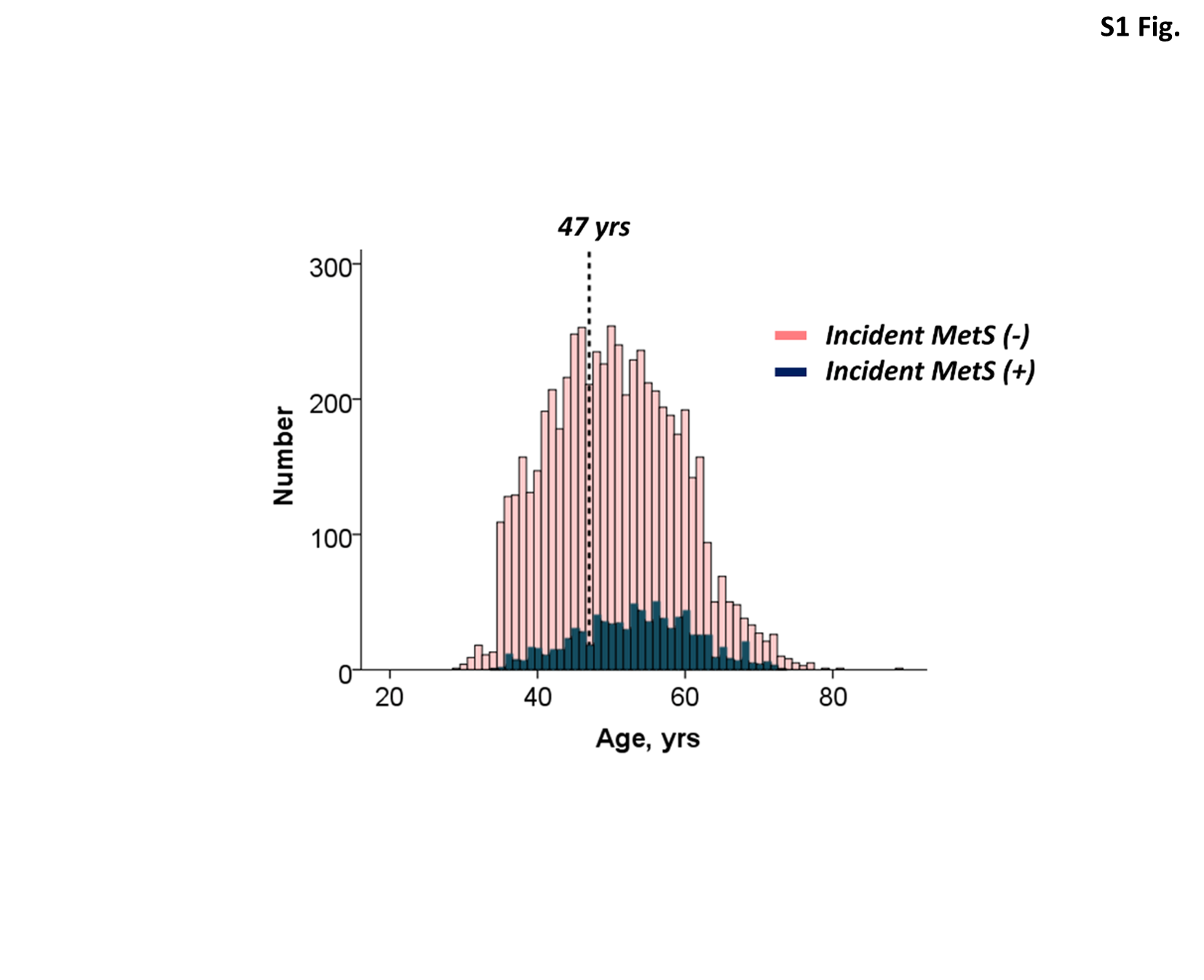

Supplement: S1 Fig — Age was dichotomized using optimal cut-off points (47 years) derived from Youden index to construct a scoring system. Pink bar: participants without MetS, dark-blue bar: participants with incident MetS; abbreviations as in Fig 1. (TIF) [file pone.0133884.s001.tif]
