# Supplementary material for: A Risk Score with Additional Four Independent Factors to Predict the Incidence and Recovery from Metabolic Syndrome: Development and Validation in Large Japanese Cohorts
Source: PLoS One. 2015 Jul 31;10(7):e0133884. doi: 10.1371/journal.pone.0133884 (PMC4521863; doi:10.1371/journal.pone.0133884)
Supplement: S1 Table — Abbreviations as in Tables 1 and 2. (DOCX) [file pone.0133884.s002.docx]

**S1 Table. Receiver-operating characteristic curve of parameters predicting incident metabolic Syndrome.**

| **Variables** | **Area under the curve** | **p value** | **Optimal cut-off** | **Sensitivity** | **Specificity** |
| --- | --- | --- | --- | --- | --- |
| Age, yrs | 0.60 | <0.001 | 47 yrs | 77 % | 40 % |
| γ-GTP, IU/L | 0.65 | <0.001 | 30 IU/L | 58 % | 64 % |
| Alkaline Phosphatase, IU/L | 0.60 | <0.001 | 200 IU/L | 70 % | 45% |
| LDL-cholesterol, mg/dL | 0.65 | <0.001 | 130 mg/dL | 64 % | 59 % |
| Hematocrit, % | 0.59 | <0.001 | 45 % | 46 % | 68% |
| Uric acid, mg/dL | 0.62 | <0.001 | 6.0 mg/dL | 43 % | 73 % |

Abbreviations as in Tables 1 and 2.
