# Supplementary material for: A Risk Score with Additional Four Independent Factors to Predict the Incidence and Recovery from Metabolic Syndrome: Development and Validation in Large Japanese Cohorts
Source: PLoS One. 2015 Jul 31;10(7):e0133884. doi: 10.1371/journal.pone.0133884 (PMC4521863; doi:10.1371/journal.pone.0133884)
Supplement: S2 Table — Reclassification was assessed using these two tables: those with subsequent MetS (Top) and those without (Bottom). Although 209 people were misclassified in the top table, 5646 people without outcome (subsequent Mets) were correctly reclassified into lower risk category. Consequently, 34% of Non-MetS population were correctly reclassified. NRI, net reclassification improvement; other abbreviations as in Table 1. (DOCX) [file pone.0133884.s003.docx]

**S2 Table. Reclassification tables for NRI calculation for subsequent MetS risk.** Reclassification was assessed using these two tables: those with subsequent MetS (*Top*) and those without (*Bottom*). Although 209 people were misclassified in the top table, 5646 people without outcome (subsequent Mets) were correctly reclassified into lower risk category. Consequently, 34% of Non-MetS population were correctly reclassified.

| Outcome (+) | Final score | | | | Up reclassified | |
| --- | --- | --- | --- | --- | --- | --- |
| Model based of 5 MetS factors |  | [0, 2%] | [2%, 9%] | [9%, 100%] | n | % |
|  | [0, 2%] | 0 | 0 | 0 | 0 | 0 |
|  | [2%, 9%] | 45 | 94 | 14 | -31 | -39 |
|  | [9%, 100%] | 0 | 178 | 1304 | -178 | -12 |

| Outcome (-) | Final score | | | | Down reclassified | |
| --- | --- | --- | --- | --- | --- | --- |
| Model based of 5 MetS factors |  | [0, 2%] | [2%, 9%] | [9%, 100%] | n | % |
|  | [0, 2%] | 0 | 0 | 0 | 0 | 0 |
|  | [2%, 9%] | 3574 | 2246 | 64 | 3510 | 62 |
|  | [9%, 100%] | 0 | 2136 | 3979 | 2136 | 35 |

NRI, net reclassification improvement; other abbreviations as in Table 1.
